# Supplementary material for: Frozen Ready-to-(h)eat Meals: Evolution of Their Quality during a Real-Time Short Shelf Life
Source: Foods. 2023 Mar 3;12(5):1087. doi: 10.3390/foods12051087 (PMC10000526; doi:10.3390/foods12051087)
Supplement: Supplementary file 1 [file foods-12-01087-s001.zip › SENSORY ANALYSIS FORM ready-to-eat SOUP.pdf]

Ready-to-(h)eat Creamy Soup Sensory Analysis Form

APPEARANCE

Orange color ____

Yellow/brown color ____

Homogeneity of color ____

Brilliance ____

Surface

Greasiness-Oiliness ____

Translucent/watery ____

Presence of mould ____

Palatable ____

Other ____

ODOR

Overall intensity odor ____

Carrot odor ____

Onion odor ____

Tomato odor ____

Potato odor ____

Grain odor ____

Pumpkin odor ____

Fresh vegetable odor ____

Spice odor ____

Oil odor ____

Broth odor ____

Metallic odor ____

Rancid ____

Fermented ____

Burnt ____

Paper/cardboard ____

Other ____

TASTE

Sweet ____

Salty ____

Sour ____

Bitter ____

Umami ____

TEXTURE

Viscosity ____

Graininess ____

Clumpiness ____

Creaminess ____

Chewiness ____

Cohesiveness ____

Solubility ____

Crunchiness ____

Adhesiveness ____

Consistency ____

Softness ____

Fat content ____

(greasiness - oiliness)

Moisture ____

(presence of a high liquid content)

TRIGEMINAL SENSATIONS

Spiciness ____

Astringency ____

Burning ____

Metallic ____

FINAL SENSATIONS

Aftertaste ____

Persistence ____

SAMPLE

DATE TASTER
