# Supplementary material for: Frozen Ready-to-(h)eat Meals: Evolution of Their Quality during a Real-Time Short Shelf Life
Source: Foods. 2023 Mar 3;12(5):1087. doi: 10.3390/foods12051087 (PMC10000526; doi:10.3390/foods12051087)
Supplement: Supplementary file 1 [file foods-12-01087-s001.zip › SENSORY ANALYSIS FORM ready-to-eat TORTELLINI.pdf]

Ready-to-(h)eat Tortellini Sensory Analysis Form

APPEARANCE

Color of pasta (Bright yellow) ____

Homogeneity of color ____

Brilliance ____

Surface of the paste

Smooth ____

Swollen rough and deformed ____

Presence of mould ____

Moisture ____

Succulent ____

Overcooked ____

Stringy ____

Meat color light brown ____

Meat color dark brown ____

Clotted cream ____

ODOR

Overall intensity odor ____

Pasta odor ____

Cooking cream odor ____

Meat odor ____

Cooked ham odor ____

Broth odor ____

Vegetable odor ____

Spice odor ____

Cheese odor ____

Oil odor ____

Rancid ____

Metallic ____

Burnt ____

Paper/cardboard ____

Other ____

TASTE

Sweet ____

Salty ____

Sour ____

Bitter ____

Umami ____

TEXTURE

Springiness ____

Hardness ____

Firmness ____

Chewiness ____

Smoothness/sliminess ____

Roughness ____

Cohesiveness ____

Solubility ____

Adhesiveness ____

Consistency ____

Graininess ____

Gumminess ____

Fat content (greasiness - oiliness) ____

Moisture ____

(presence of a high liquid content)

Overcooked ____

TRIGEMINAL SENSATIONS

Spiciness ____

Astringency ____

Burning ____

Metallic ____

FINAL SENSATIONS

Aftertaste ____

Persistence ____

SAMPLE

DATE TASTER
