# Supplementary material for: Frozen Ready-to-(h)eat Meals: Evolution of Their Quality during a Real-Time Short Shelf Life
Source: Foods. 2023 Mar 3;12(5):1087. doi: 10.3390/foods12051087 (PMC10000526; doi:10.3390/foods12051087)
Supplement: Supplementary file 1 [file foods-12-01087-s001.zip › Triangle test form.pdf]

Triangle test (ISO 4120:2021)

Position X – TEST 1

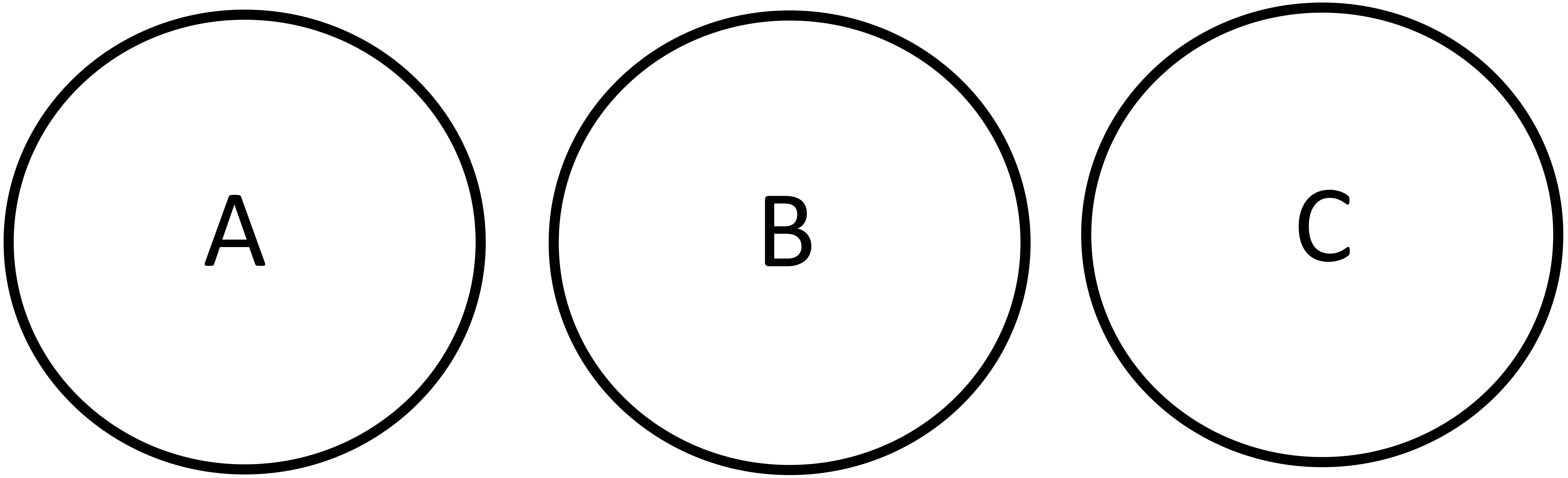

Indicate the different sample ________

Date ____________ Taster _____________

Triangle test (ISO 4120:2021)

Position X – TEST 2

D

E

F

Indicate the different sample ________

Date ____________

Taster _____________
